# Nucleolar NOL9 regulated by DNA methylation promotes hepatocellular carcinoma growth through activation of Wnt/β-catenin signaling pathway

**DOI:** 10.1038/s41419-025-07393-7

**Published:** 2025-02-15

**Authors:** Xiyao Chen, Xin Song, Xingrong Zheng, Tinglin Qian, Boxiang Zhang, Lina Wu, Qinghai Lian, Jia Chen, Qiumin Luo, Wenxiong Xu, Liang Peng, Chan Xie

**Affiliations:** 1 Department of Infectious Diseases, The Third Affiliated Hospital of Sun Yat-Sen University, Guangzhou, Guangdong China; 2 Guangdong Key Laboratory of Liver Disease Research, The Third Affiliated Hospital of Sun Yat-Sen University, Guangzhou, China; 3 Department of Cell-Gene Therapy Translational Medicine Research Center, The Third Affiliated Hospital of Sun Yat-Sen University, Guangzhou, China

**Keywords:** Cancer therapeutic resistance, Liver cancer

## Abstract

Ribosome biogenesis (RiboSis) and ribosomal stress are critical in tumor progression, positioning RiboSis as a promising therapeutic target for cancer treatment and for overcoming drug resistance. In this study, we examined the role of RiboSis in the progression from hepatitis B virus (HBV) infection to HBV-related hepatocellular carcinoma (HCC), focusing specifically on nucleolar protein 9 (NOL9) and its influence on HCC pathogenesis and therapeutic response. Our findings showed that NOL9 was significantly upregulated in HCC tissues, correlating with larger tumor sizes and more advanced pathological grades. High levels of NOL9 expression were associated with unfavorable prognosis in both the TCGA-LIHC and our HCC cohorts. Functional assays indicated that NOL9 regulated HCC cell proliferation and apoptosis; specifically, NOL9 knockdown inhibited cell proliferation and promoted apoptosis, while overexpression enhanced these processes. In vivo studies confirmed that NOL9 depletion reduced tumor growth. Mechanistically, NOL9 expression was regulated by DNA methylation and the transcription factor ZNF384. Our DNA methylation analysis revealed an inverse correlation between NOL9 expression and methylation at specific CpG sites, implicating DNMT1 in its epigenetic regulation. Additionally, NOL9-mediated cell proliferation was dependent on activation of the Wnt/β-catenin signaling pathway. This study highlights the multifaceted role of NOL9 in HCC pathogenesis, underscoring its potential as a diagnostic biomarker and therapeutic target.

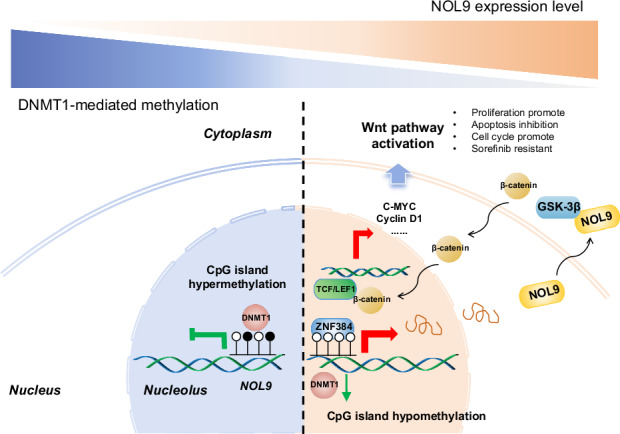

## Introduction

Hepatocellular carcinoma (HCC) is the most common primary liver cancer, accounting for 75-85% of cases globally [1]. Despite advancements in care, it remains highly lethal, with a five-year survival rate below 20% worldwide [2]. Survival rates vary significantly based on the stage of diagnosis and healthcare access, with early-stage patients faring much better than those with advanced disease [3, 4]. These alarming outcomes highlight the urgent need to explore the molecular mechanisms underlying HCC progression to enhance diagnostic and therapeutic strategies.

Cancer cells, known for their unlimited replicative potential and elevated metabolic demands, depend heavily on increased global protein synthesis for rapid proliferation [5]. Ribosome biogenesis (RiboSis), a multistep process crucial for protein synthesis, plays a central role in this proliferation [6]. This process involves the coordinated activities of RNA polymerases, ribosomal proteins, and various trans-acting factors [7, 8]. Emerging evidence suggests that RiboSis is not only essential for tumor growth but also provides key prognostic and diagnostic insights in HCC [9–12]. Several risk factors, including hepatitis B virus (HBV) [13–15] and hepatitis C virus (HCV) infections [16, 17], are known to enhance RiboSis and promote tumorigenesis. These factors stimulate ribosomal RNA (rRNA) transcription via mechanisms such as p53 signaling or RNA polymerase I activation, leading to increased ribosome production and cancer cell proliferation [14, 15]. The dysregulation of RiboSis offers potential therapeutic targets, making its inhibition a promising strategy for combating HCC, particularly in advanced stages.

Our research focuses on nucleolar protein 9 (NOL9), a 5’-polynucleotide kinase involved in RiboSis [18]. NOL9, part of a nucleolar complex with PELP1, TEX10, and LAS1, plays a key role in pre-rRNA cleavage and 28S rRNA maturation, promoting efficient RiboSis [19]. A recent pan-oncogenic analysis identified NOL9 as one of the top 10 RiboSis genes most likely to undergo amplification or deletion [6]. However, despite these insights into its role in RiboSis and tumorigenesis, its impact on HCC pathogenesis remains unclear.

In this study, we aim to clarify NOL9’s role in HCC cell proliferation and tumor development, emphasizing the epigenetic mechanisms driving its aberrant expression. Our research highlights the epigenetic activation of oncogenic NOL9, mediated by DNA methylation of CpG islands within its promoter region.

## Materials and methods

### Patient selection and specimen collection

A cohort of 56 HCC patients who underwent interventional surgical procedures at the Third Affiliated Hospital of Sun Yat-sen University between 2017 and 2019 provided tissue samples for this study. All patients received postoperative pathological validation confirming their HCC diagnosis. The study’s design and execution were approved by the Ethics Committee of the Third Affiliated Hospital of Sun Yat-sen University (II2023-284-01).

### Data acquisition and analytical procedures

#### TCGA database analysis

We obtained RNA-sequencing data (FPKM format) and clinical records for 50 normal samples and 374 HCC patients from the Liver Hepatocellular Carcinoma (LIHC) project in The Cancer Genome Atlas (TCGA) database.

#### ROC curve generation

Using the R package “ROCR,” we constructed Receiver Operating Characteristic (ROC) curves.

#### Differential expression analysis

The R package “limma” was used to compare gene expression between groups, defined by the median NOL9 expression level. Genes with |logFoldChange| > 0.5 and an adjusted p-value (adj.P.Val) < 0.05 were considered differentially expressed.

#### Functional enrichment analysis

We employed the “clusterProfiler” R package for Gene Ontology (GO) and Kyoto Encyclopedia of Genes and Genomes (KEGG) enrichment analyses of the differentially expressed genes (DEGs) related to NOL9 expression. The top 30 terms were selected based on adj.P.Val < 0.05 and a gene count > 2.

#### Correlation analysis

We analyzed the correlation between NOL9 DNA methylation and its transcript expression in the TCGA-LIHC dataset. Kaplan-Meier (KM) survival analyses were conducted using the Survival and Survminer R packages to evaluate the prognostic impact of NOL9 DNA methylation/expression in HCC patients.

#### Gene expression profiling interactive analysis (GEIPA)

We analyzed the correlation between NOL9 and ZNF384 mRNA expression using the GEIPA database (http://gepia.cancer-pku.cn/).

#### Transcription factor binding site prediction

The JASPAR software was used to predict transcription factor binding sites.

### ssGSEA enrichment scores of ribosome biogenesis gene Sets

We downloaded gene expression data from the GEO database, including GSE83148 with 127 hepatitis B cases, GSE17548 with 10 HBV-related HCC cases, and GSE14520 with 160 HBV-related HCC cases. ssGSEA enrichment scores were calculated using the “GSEABase” and “limma” R packages.

For each sample, we normalized the gene expression data before calculating the ssGSEA enrichment scores using predefined gene sets (GOPB_ribosome_biogenesis and GOBP_regulation_of_ribosome_biogenesis) from the MSigDB database. The scores were based on comparing the expression levels of the gene set members with background gene sets.

To compare HBV and HBV-related HCC, we applied the Wilcoxon rank-sum test to assess the statistical significance of ssGSEA enrichment scores between the groups. A similar analysis was performed using the TCGA-LIHC dataset, comparing cancer tissues and adjacent non-tumor tissues in HCC.

All statistical analyses were performed using R software, with a P-value of <0.05 considered statistically significant.

### Cell culture and proliferation assays

Human HCC cell lines, including HepG2, Huh7, and HEK293T, were obtained from the Hepatology Laboratory of the Third Affiliated Hospital of Sun Yat-sen University. Cells were cultured in DMEM supplemented with 10% FBS and 1% penicillin/streptomycin. All the cell lines were confirmed to be free of mycoplasma contamination, and their identities were verified through STR analysis.

#### CCK-8 assay

Cells (1000/well) were seeded into 96-well plates. After cell attachment (24 h), the medium was replaced with CCK-8-containing media. Following a 1-hour incubation at 37 °C, absorbance was measured at 450 nm using a microplate reader.

#### EdU assay

Cells (2 × 10^4^/mL) were seeded into 48-well plates and subjected to the EdU Cell Proliferation Kit following the manufacturer’s instructions. After Hoechst staining for 3 min, cellular imaging was performed using fluorescence microscopy.

#### Colony formation assay

Cells (1000/well) were seeded into six-well plates. After 14–20 days of culture, cells were fixed and stained, and colonies containing more than 50 cells were counted.

### Immunohistochemistry analysis

A cohort of 56 HCC patients was established, from whom cancerous and para-cancerous liver tissues were collected, along with clinical and survival data. Additionally, liver cancer tissue microarrays were obtained from Superchip (# HLivH180Su08, Shanghai, China). Each array contained adjacent HCC and primary HCC tissues from 90 cases. Comprehensive survival data and biochemical indicators were also available.

Paraffin-embedded tissue sections were deparaffinized in an oven, and endogenous peroxidase activity was inhibited using 3% hydrogen peroxide. Antigen retrieval was conducted using EDTA repair solution under high pressure and temperature. Tissue sections were outlined using a histochemical pen, approximately 0.5 cm from the tissue margin. The primary antibody against NOL9 (1:200, #abs139241, Absin, China) was applied according to the manufacturer’s protocol. After primary antibody incubation and washing, sections were treated with the appropriate secondary antibodies. Microscopic imaging was performed post-staining.

#### Scoring methodology

Ten random fields of view were selected for evaluation. Immunohistochemical staining intensity was categorized into four levels: no staining (1), weak staining (2), moderate staining (3), and strong staining (4). The proportion of positively stained cells was graded as follows: <25% (1), 25–50% (2), 50–75% (3), and >75% (4). The final score for each sample was calculated as the product of staining intensity and the percentage of positively stained cells.

### Western blot analysis

HCC tissues and cells were lysed using RIPA lysis buffer. Proteins were separated via SDS-PAGE and transferred onto polyvinylidene fluoride (PVDF) membranes. After blocking, membranes were probed overnight at 4 °C with primary antibodies: NOL9 (1:1000, # ab140597, Abcam, UK), GAPDH (1:3000, # ab8245, Abcam, UK), β-tubulin (1:5000, # A12289-50, ABclonal, China), Rb (1:1000, # 9309S, CST, USA), P-Rb (1:1000, # 8516 T, CST, USA), CDK6 (1:1000, # PA5-27978, ThermoFisher, USA), Cyclin D1 (1:1000, # 2978 T, CST, USA), β-catenin (1:1000, # 9562S, CST, USA), LaminA/C (1:25000, # 10298-1-AP, Proteintech, China), and GSK-3β (1:1000, # 12456 T, CST, USA). Secondary antibodies, either goat anti-rabbit IgG-HRP (1:5000, # E030120-01, SaiguoTech, Guangzhou, China) or goat anti-mouse IgG-HRP (1:5000, # E030110-01, SaiguoTech, Guangzhou, China), were applied for 1 h at room temperature. Protein bands were visualized using the Excellent Chemiluminescent Substrate detection kit and quantified via grayscale analysis using ImageJ (version 1.8.0).

### Quantitative real-time PCR (qRT-PCR)

Total RNA was isolated from 1 × 10^6^ cells using the RNA Quick Purification Kit. Then, 1 µg of RNA was reverse transcribed into cDNA using the Fast Reverse Transcription Kit (5 × Mix with gDNA Remover). qRT-PCR was conducted using the Takara TB Green Premix Ex Taq II, following a 3-step cycling protocol and the manufacturer’s guidelines. Primer sequences are listed in Supplementary Table 2.

### Cell transfection

Cells were seeded in six-well plates and, upon reaching 60% confluence, were transfected with either gene-specific siRNA or control siRNA (Ribobio, Guangzhou, China) using Lipofectamine 3000 (Invitrogen, MA, USA). After 24 hours, qRT-PCR confirmed target gene knockdown. At 48 h, Western blotting assessed knockdown efficiency. Only cultures showing an 80% or greater reduction in target gene mRNA and protein expression, compared to controls, proceeded to subsequent experiments.

### Lentivirus package and stable cell lines

For the generation of stable NOL9-overexpressing or knockdown cell lines, 293 T cells were used to package recombinant lentiviruses containing either the NOL9 gene or specific shRNAs targeting NOL9 (shNOL9-#1, shNOL9-#2). A lentiviral vector with a non-specific sequence served as a control. Target cells were incubated in serum-rich medium supplemented with viral particles and polybrene. Stably transfected cells were selected using puromycin (InvivoGen, France).

### Methylation-specific PCR (MSP)

Genomic DNA was isolated from 5 × 10^6^ cells using the Promega Wizard Genomic DNA Purification Kit, following the manufacturer’s instructions. Bisulfite conversion of the DNA was then performed using EpiTect Bisulfite Kits (Qiagen). Quantitative real-time PCR was used to assess the methylation status of the NOL9 promoter regions. Primer sequences are provided in Supplementary Table 2.

### Flow cytometry for cell cycle and apoptosis analysis

For cell cycle analysis, cells were serum-starved for 24 h, collected, fixed in 70% ethanol overnight at 4 °C, and stained with propidium iodide (PI) before flow cytometry. Apoptosis was evaluated using PI and Annexin V-APC staining, following the manufacturer’s protocol (KeyGEN, China).

### Luciferase reporter assays

Cells were plated in 12-well plates and, after 24 h, transfected with the indicated plasmids. After 36 h, cell lysates were analyzed for luciferase activity using the Dual-Luciferase Reporter Assay System (Promega, # E1910), following the manufacturer’s instructions.

### Chromatin immunoprecipitation (ChIP)

Cells were cross-linked with 1% formaldehyde for 10 min, and the reaction was terminated with glycine. Lysates were sonicated to produce chromatin fragments, which were then immunoprecipitated with the ZNF384 antibody (# ab176689, Abcam, UK). IgG was used as a negative control. ChIP primer sequences are listed in Supplementary Table 2.

### TOP/FOP-flash Wnt reporter analysis

HCC cells were cultured in 12-well plates at approximately 50% confluency for TOP/FOP-flash Wnt reporter assays. Cells were co-transfected with OE-NOL9/si-NOL9, TOP/FOP-flash plasmid (Beyotime, Shanghai, China), and renin luciferase reporter plasmid (pRL-TK). After 48 h of transfection, cells were analyzed using the dual-luciferase assay kit (Promega, # E1910). Luciferase activity was measured using a microplate reader.

### In vivo tumorigenesis assay

The tumorigenesis assay was conducted using 4 to 6-week-old BALB/c nude mice. Each mouse was injected subcutaneously with 5 × 10^6^ cells on the right hind flank, with three mice per group. Tumor size was calculated using the formula: volume (cm^3) = (length × width²)/2. After the experiment, mice were humanely euthanized, and tumors were surgically removed and weighed. All procedures were approved by the Animal Experimentation Ethics Committee of The Third Affiliated Hospital of Sun Yat-sen University, and appropriate care was provided by the Laboratory Animal Center Staff.

### RNA-sequencing analysis

Total RNA was extracted from control HCC cells and NOL9-knockdown HCC cell lines using Trizol, with three biological replicates per condition. RNA samples were sent to Guangzhou Kidio Biotechnology Co., Ltd (Guangzhou, China) for RNA sequencing (RNA-seq) using the Illumina platform. Data analysis was performed on Kidio’s cloud platform, OmicShare.

### Nuclear-cytoplasmic fractionation

Cells in six-well plates at 70–80% confluence were transfected with plasmids. After 48 h, the medium was discarded, and the cells were washed twice with PBS. Cells were collected using trypsin-EDTA, centrifuged at 500 × g for 5 min, and washed once with cold PBS. Two million cells were transferred to a 1.5 mL microcentrifuge tube and pelleted by centrifugation at 500 × *g* for 2–3 min. The supernatant was carefully removed, leaving the cell pellet as dry as possible, before adding 200 μL of ice-cold CER I and vortexing for 15 s.

### Statistical analysis

Data from the TCGA and GEO databases were integrated and analyzed using R software (version 4.1.0) and GraphPad Prism (version 9.0). In vitro experiments were performed at least three times, with results presented as mean ± standard deviation. Differences between two groups were evaluated using Student’s t-test, while associations between clinical attributes and NOL9 expression were determined using the Chi-squared test. For comparisons involving three or more groups, one-way or two-way ANOVA was used for parametric data, and Kruskal–Wallis tests for non-parametric data. Pearson’s correlation was used to assess gene correlations. Hazard ratios were estimated with a 95% confidence interval. A two-tailed *P*-value ≤ 0.05 was considered statistically significant.

## Results

### NOL9 is upregulated in HCC and correlates with patient survival

**Fig. 1:**
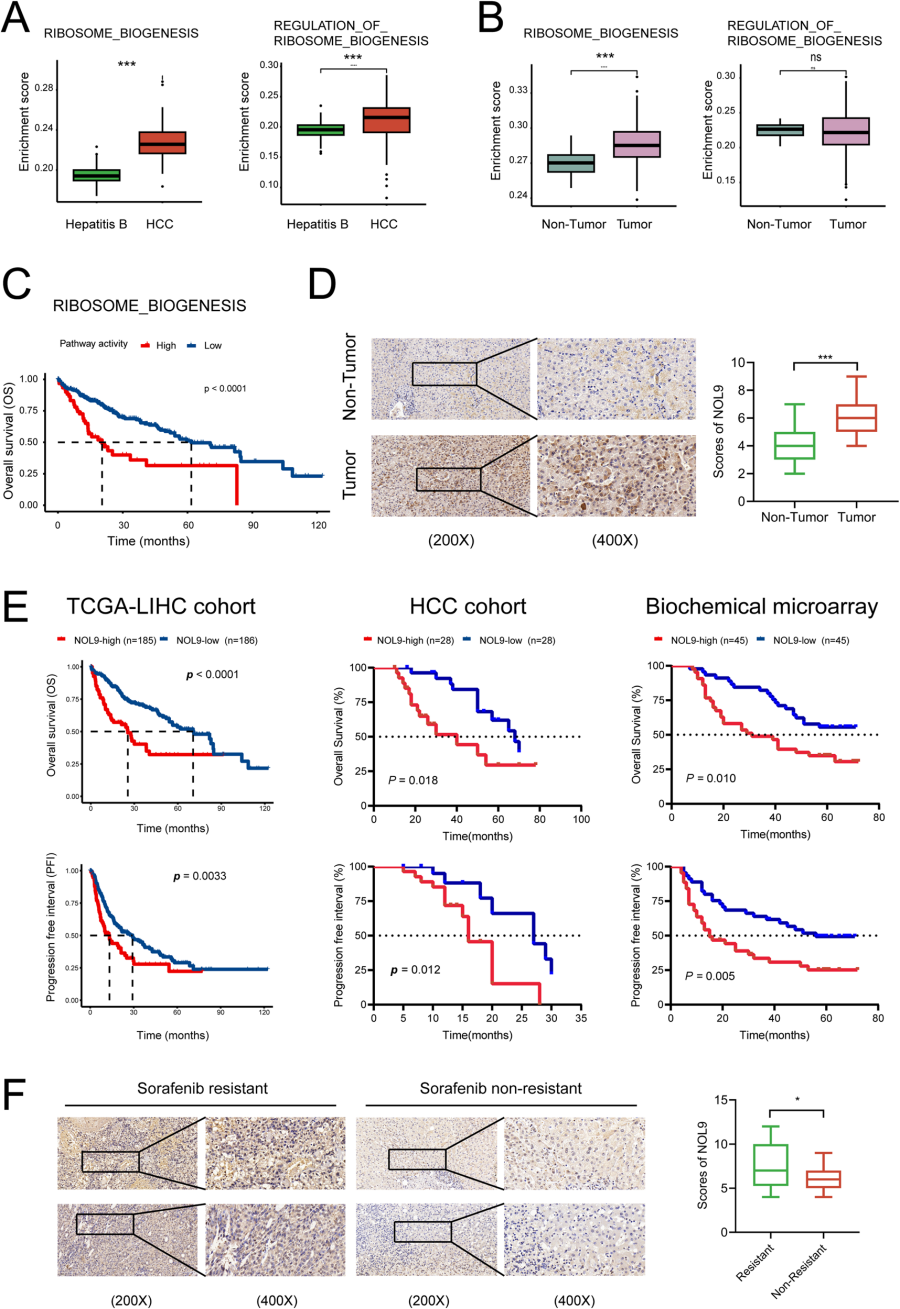
NOL9 is upregulated in HCC and correlates with patient survival. **A** Sequencing data for patients with hepatitis B virus infection and hepatitis B-related HCC were retrieved from the GEO dataset. The enrichment scores of the Ribosome Biogenesis and Regulation of Ribosome Biogenesis pathways in the two patient populations were analyzed. **B** In patients with hepatitis B-related HCC, the enrichment scores of these two pathways were assessed in cancerous tissues versus adjacent non-cancerous tissues. **C** HCC patients were stratified into high-expression and low-expression groups based on the median enrichment score. Survival differences between the two groups were then analyzed. **D** Immunohistochemistry (IHC) analysis of NOL9 expression in 56 paired HCC and tumor-adjacent normal tissue samples. NOL9 protein levels were significantly upregulated in HCC tumor samples compared to normal liver tissues. **E** Kaplan–Meier survival curve analysis showing the correlation between NOL9 expression and patient survival. **F** IHC analysis of NOL9 expression in 13 sorafenib-resistant HCC tumor samples. NOL9 protein levels were significantly upregulated in sorafenib-resistant samples compared to sorafenib-sensitive samples. *p < 0.05, ***p < 0.001. T tumor, N non-tumor.

Ribosome biogenesis plays a critical role in protein synthesis, and its dysregulation is closely associated with HCC development and progression. Figure 1A, B show that ribosome biogenesis scores increase from hepatitis B to HBV-related HCC samples, with a more pronounced elevation in cancerous tissues compared to adjacent non-cancerous tissues. This suggests that targeting ribosome biogenesis may offer new strategies for HCC therapy. Prognostic analysis revealed that HCC patients with higher ribosome biogenesis scores exhibited lower survival rates compared to those with lower scores (Fig. 1C).

Our previous research identified a significant increase in NOL9 transcriptional levels in HCC compared to normal tissues [20]. In the current study, we first analyzed the clinical significance of NOL9 using the TCGA-LIHC cohort. Clinical data indicated that patients with higher NOL9 expression exhibited larger tumor sizes and more advanced pathological grades (moderate differentiation) (Supplementary Fig. 1A). ROC curve analysis revealed that NOL9 effectively distinguished HCC samples from normal ones (AUC = 0.817) (Supplementary Fig. 1B).

To assess the impact of multiple factors on survival, we included age, tumor TNM staging, gender, stage, and other clinical pathological factors, along with NOL9 expression, for univariate Cox analysis. The p-values for tumor size (T), node status (N), metastasis (M), stage, and NOL9 were all less than 0.05. These factors were then included in a multivariate Cox analysis, which showed that the p-values for T stage, M stage, and NOL9 were all less than 0.1 (Supplementary Fig. 1C). Based on these results, a nomogram was constructed using NOL9, T stage, and M stage to estimate the 1-, 3-, and 5-year survival rates of HCC patients (Supplementary Fig. 1D). A calibration curve was also constructed to compare the predicted survival rates with actual survival rates (Supplementary Fig. 1E).

To validate this, we examined NOL9 expression in our own set of carcinoma and adjacent tissue samples. Both mRNA and protein levels of NOL9 were significantly elevated in seven paired fresh HCC tissues (Supplementary Fig. 1F). Extending the analysis to a larger set of paraffin-embedded tissues confirmed NOL9’s overexpression in HCC (Fig. 1D). KM curve analysis showed that high NOL9 expression was correlated with poor prognosis in the TCGA-LIHC cohort, our HCC cohort, and the biochemical microarray cohort (Fig. 1E). We also analyzed samples from 13 patients exhibiting poor sorafenib efficacy. These samples displayed a significant increase in NOL9 protein levels compared to those with a good response (P = 0.025) (Fig. 1F).

To further verify the clinical implications of NOL9 in our cohort, patients were categorized into high-expression (28 samples) and low-expression (28 samples) NOL9 groups based on median staining scores. Clinical data indicated that the high NOL9 expression cohort also exhibited larger tumor sizes and more advanced pathological grades (Table 1), consistent with TCGA results (Table 2).

**Table 1:** Relationship between NOL9 protein expression and clinical characteristics in our hospital HCC cohort.

Clinical characteristics	Groups		*P* value
	High expression group (*n* = 28)	Low expression group (*n* = 28)	
Age (Year)	56.3 ± 13.0	55.0 ± 10.1	0.67
Gender			0.75
Male	22	21	
Female	6	7	
HBV			0.64
Negative	3	2	
Positive	25	26	
Liver cirrhosis			0.31
Yes	21	24	
No	7	4	
Serum AFP			0.79
<20 μg/ml	12	13	
≥20 μg/ml	16	15	
TBIL (μmol/L)	12.8 (10.0~18.2)	11.9 (10.9~15.6)	0.82
Recurrence			**0.04**
Yes	13	6	
No	15	22	
Pathological differentiation			**0.02**
Minor differentiation	13	21	
Moderate differentiation	15	7	
Tumor size (mm)			**0.03**
T1 & T2	10	18	
T3 & T4	18	10	
Tumor number			0.36
Multiple (≥2)	9	6	
Single	19	22	
Metastasis			**<0.01**
Yes	15	5	
No	13	23	
BCLC stage			**<0.01**
A stage	9	20	
B stage	4	4	
C stage	15	4

**Table 2:** Relationship between NOL9 protein expression and clinical characteristics in TCGA-LIHC.

Clinical characteristics	Groups		*P* value
	High expression group (*n* = 185)	Low expression group (*n* = 186)	
Tumor size			**0.03**
T1 & T2	130	148	
T3 & T4	55	38	
Pathological differentiation			**<0.01**
G1 & G2	103	130	
G3 & G4	81	55	

### NOL9 modulates HCC cell proliferation and apoptosis

**Fig. 2:**
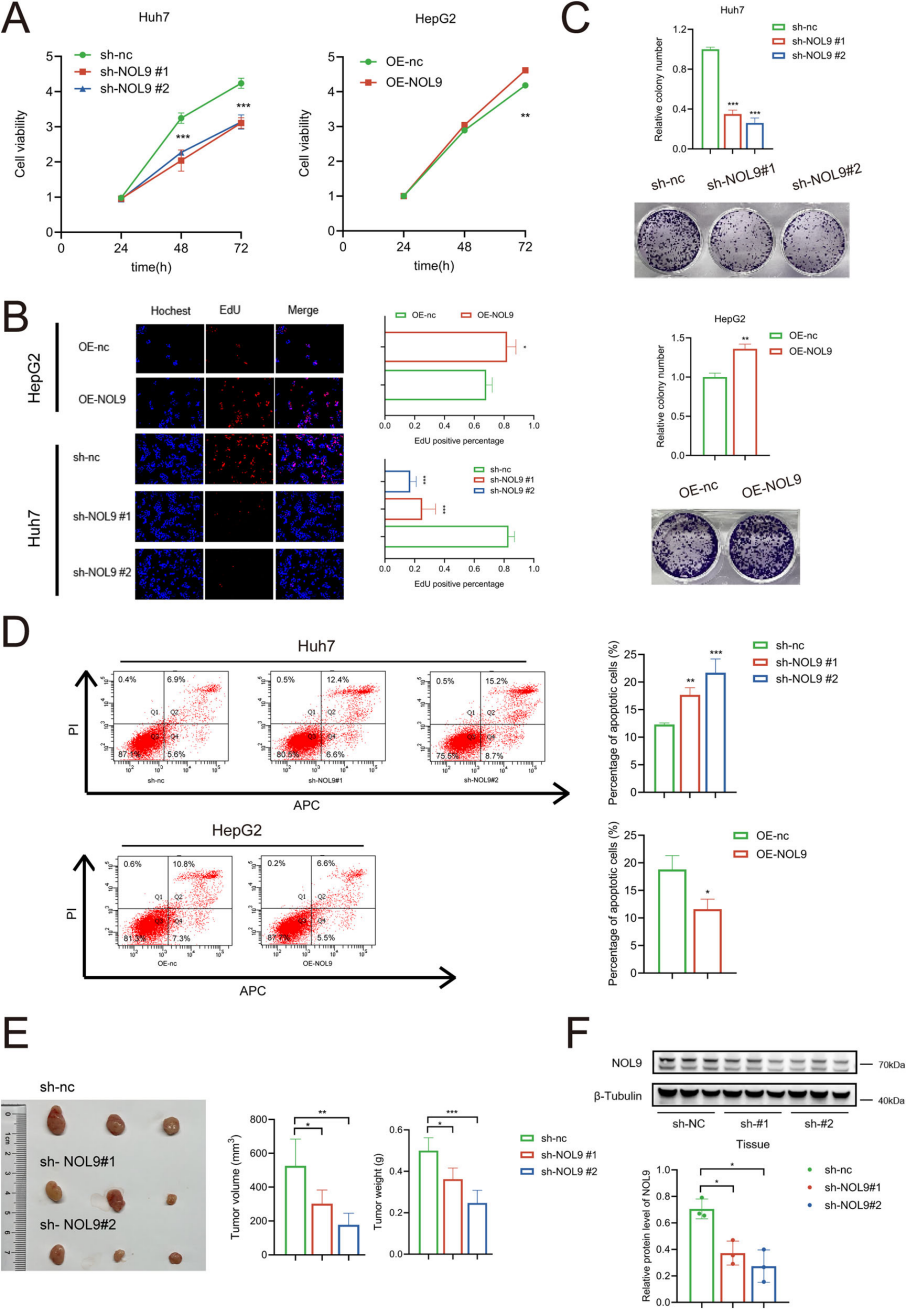
NOL9 modulates HCC cell proliferation and apoptosis. **A** CCK-8 assay showing the proliferation of HCC cells. **B** EdU assay showing the proliferation of HCC cells. **C** Clonogenic assay showing the colony formation ability of HCC cells. **D** Flow cytometry assay showing the percentage of apoptotic HCC cells. **E** Huh7 cells with stable NOL9 knockdown were subcutaneously injected into the right flanks of nude mice (*n* = 3 per group). Tumor volumes and tumor weights on day 30 post-transplantation were measured. **F** Western blotting analysis of NOL9 expression. *p < 0.05, **p < 0.01, ***p < 0.001.

To elucidate NOL9’s functional significance in HCC, we introduced two distinct shRNAs into the NOL9-high cell line Huh7 (Supplementary Fig. 2A, B). The efficacy of this knockdown was confirmed via western blotting and qRT-PCR (Supplementary Fig. 2B). Both sh-NOL9#1 and sh-NOL9#2, which showed robust knockdown efficiency, were used in subsequent experiments. Conversely, in the NOL9-low expressing cell line HepG2, NOL9 was overexpressed, as verified by western blotting and qRT-PCR (Supplementary Fig. 2C). NOL9 suppression resulted in a significant reduction in cellular proliferation and clonogenic survival (Fig. 2A–C) and a concomitant increase in apoptosis (Fig. 2D). In contrast, NOL9 overexpression promoted cellular proliferation and clonogenic survival (Fig. 2A–C) while significantly reducing apoptosis (Fig. 2D). To investigate the effect of NOL9 on HCC proliferation in vivo, nude mice were injected with Huh7 cells either depleted of NOL9 or transduced with sh-ctrl. Compared to the control xenografts, which showed tumor progression, the xenografts containing NOL9-depleted HCC cells displayed a significant reduction in tumor growth (Fig. 2E). The protein expression of NOL9 in these xenografts was confirmed via western blotting (Fig. 2F). Collectively, these findings highlight NOL9’s dual role: its inhibition curtails cell growth, while its overexpression fosters it.

### Effect of NOL9 on the Cell Cycle and Ribosomal RNA Processing

**Fig. 3:**
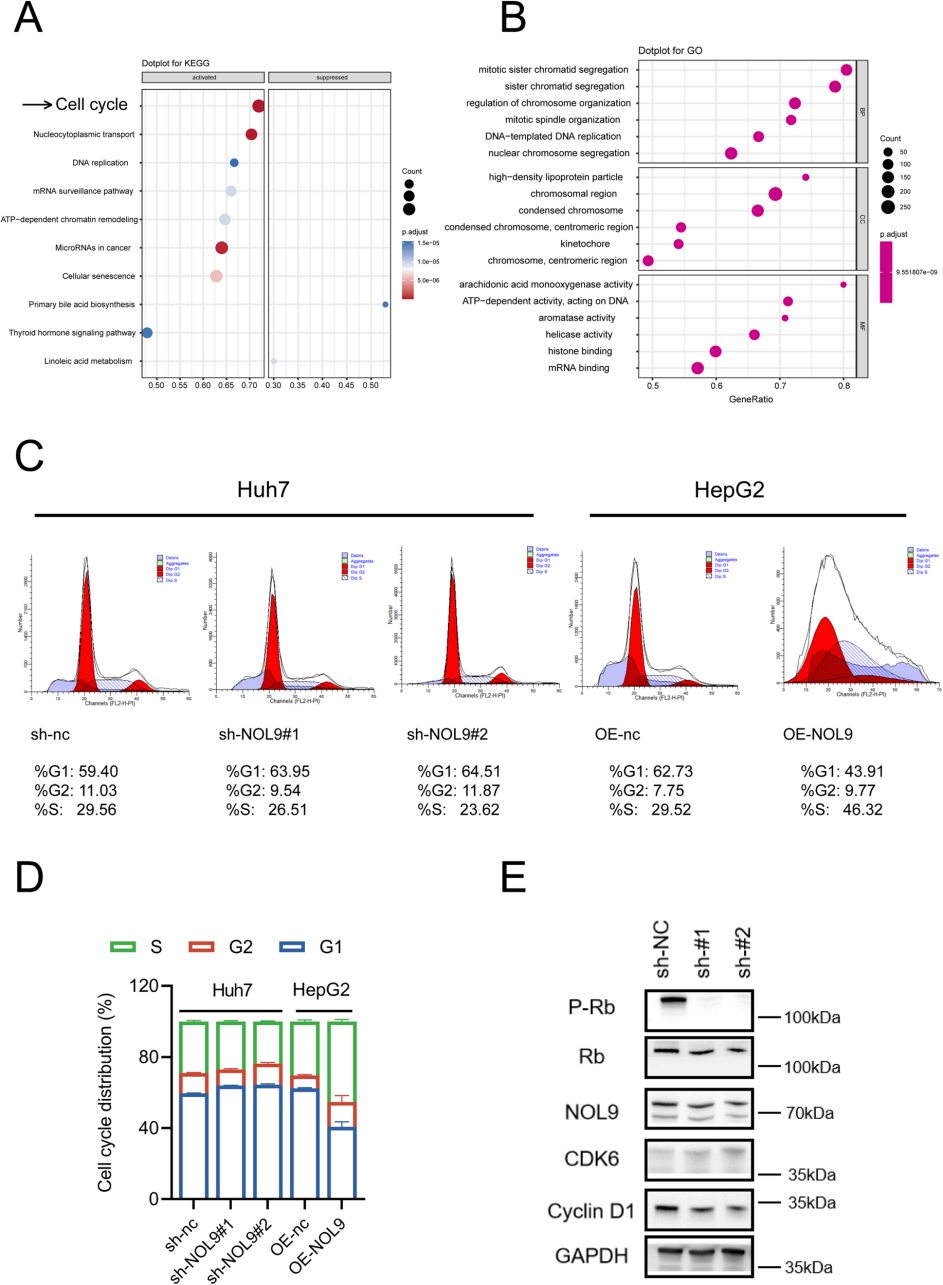
Effect of NOL9 on the HCC Cell Cycle. **A** KEGG analysis of NOL9-associated genes in the TCGA HCC dataset. **B** GO analysis of NOL9-associated genes in the TCGA HCC dataset. **C**, **D** Flow cytometric analysis of the cell cycle distribution in HCC cells. **E** Western blotting analysis of P-Rb, Rb, NOL9, CDK6, and Cyclin D1 expression.

KEGG and GO analyses of NOL9-associated genes from the TCGA-LIHC dataset revealed NOL9’s critical role in cell cycle regulation, potentially influencing processes such as chromosome segregation, nuclear division, DNA replication, and cell cycle checkpoints (Fig. 3A, B). We further investigated how modulating NOL9 levels affects the HCC cell cycle. Overexpression of NOL9 caused a notable shift in cell cycle dynamics, characterized by an 18% reduction in the G1 phase cell population. Conversely, NOL9 downregulation led to an 8% increase in G1 phase cells (Fig. 3C, D). These findings suggest that NOL9 promotes HCC cell proliferation and colony formation by facilitating the G1/S cell cycle transition and inhibiting apoptosis. Western blotting showed that NOL9 downregulation led to a reduction in Cyclin D1 protein expression and Rb protein phosphorylation, with only a slight effect on CDK6 protein expression (Fig. 3E). Ribosome biogenesis and the cell cycle are closely interconnected, as ribosome production is essential to meet the cellular growth and division demands of the cycle. NOL9 plays a pivotal role in pre-rRNA cleavage and the maturation of 28S rRNA [19]. In our study, increased NOL9 expression resulted in reduced pre-rRNA levels and elevated 28S rRNA levels, whereas NOL9 knockdown led to an accumulation of pre-rRNA and a corresponding decrease in 28S rRNA levels (Supplementary Fig. 2D).

### Transcription factor ZNF384 regulate the expression of NOL9

**Fig. 4:**
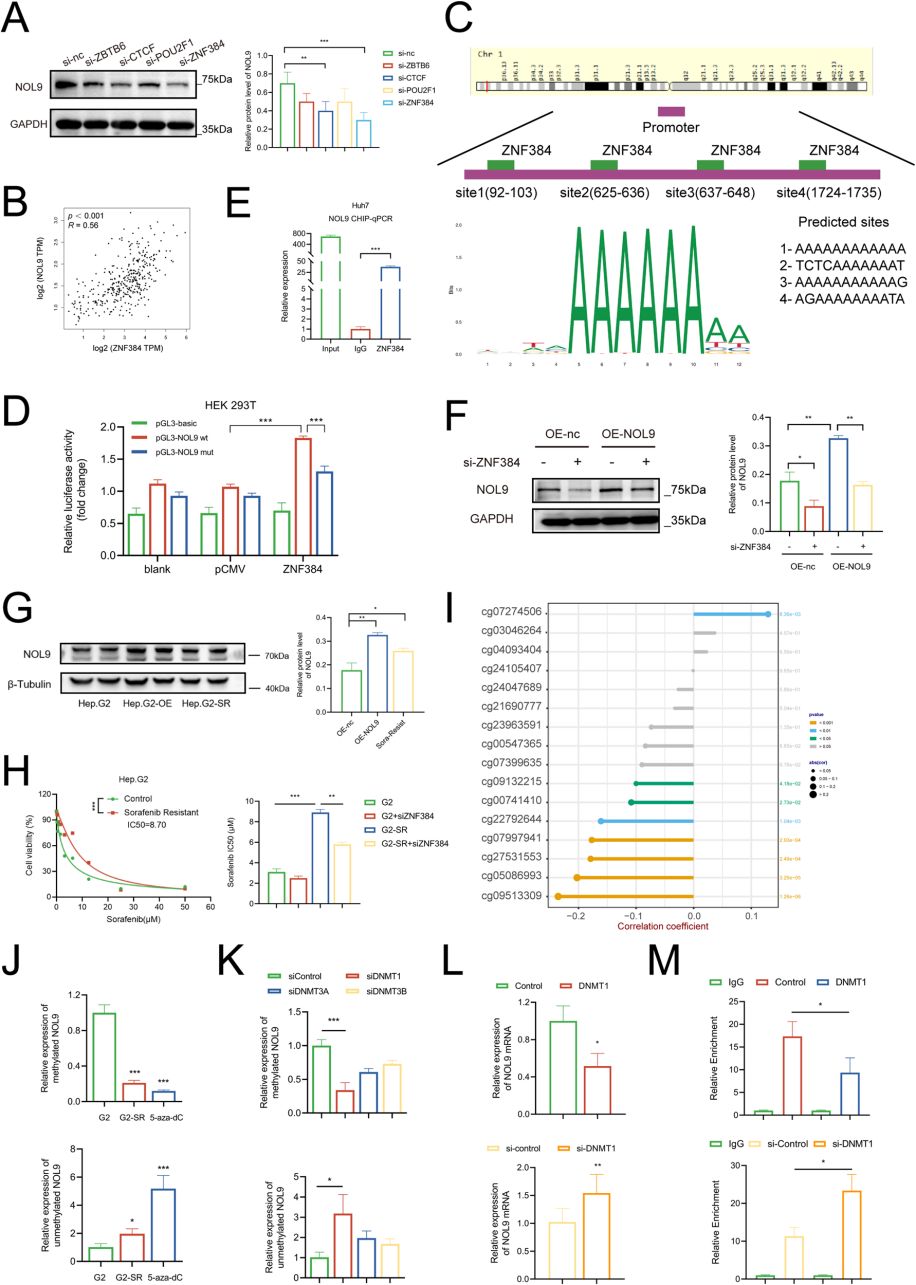
DNA methylation and ZNF384 regulate the expression of NOL9. **A** siRNAs targeting four transcription factors (predicted by the JASPAR database) were transfected into Huh7 cells. Western blotting analysis was performed to assess NOL9 expression. **B** In the TCGA-LIHC dataset, NOL9 and ZNF384 mRNA levels were positively correlated. **C** Predicted ZNF384-binding sites in the promoter region of NOL9. **D** Luciferase reporter plasmids carrying the NOL9 promoter region were co-transfected into HEK293T cells with ZNF384 plasmids. Relative luciferase activity in HEK293T cells was determined. **E** ChIP assay showing the enrichment of ZNF384 on the NOL9 promoter region. **F** Western blotting analysis of NOL9 expression in stably high-NOL9-expressing cells after ZNF384 knockdown. **G** Western blotting analysis of NOL9 expression in sorafenib-resistant cells. **H** CCK8 analysis of cell proliferation in sorafenib-resistant cells treated with sorafenib after ZNF384 knockdown. **I** Methylation levels at different loci in the NOL9 gene. **J** Methylation PCR analysis of HepG2, sorafenib-resistant HepG2, and HepG2 cells treated with 5-aza-DC. **K** Cells were transfected with siRNA targeting DNMT1, DNMT3A, and DNMT3B. Nontargeting siRNA was used as a control (siControl). Methylation PCR analysis was performed on the collected cells. **L** qRT-PCR analysis of NOL9 expression after DNMT1 overexpression or knockdown. **M** ChIP-PCR assay showing ZNF384 enrichment on the NOL9 promoter region after DNMT1 overexpression or knockdown.

We then explored the mechanism underlying the high expression of NOL9 in HCC tissues, particularly in sorafenib-resistant HCC tissues. It is well known that transcription factors are essential for gene expression. Leveraging the JASPAR database, we conducted a bioinformatics analysis of the NOL9 promoter region to predict potential transcription factor binding sites. After transfecting siRNAs targeting the top four transcription factors into HCC cells, western blotting and RT-qPCR analyses revealed that ZNF384 knockdown had the most significant impact on NOL9 expression (Fig. 4A and Supplementary Fig. 3A), suggesting that ZNF384 upregulates NOL9 in HCC cells. Additionally, a positive correlation was observed between NOL9 and ZNF384 mRNA levels in HCC (Fig. 4B).

Bioinformatics analysis predicted potential ZNF384 binding sites within the NOL9 promoter region. Given the continuous adenine sequence at site 1, we focused on verifying binding site 2 (TCTCAAAAAAAT) (Fig. 4C). Dual-luciferase reporter assays and ChIP-qPCR confirmed ZNF384’s interaction with the NOL9 promoter at binding site 2. Specifically, co-transfection of the wild-type NOL9 promoter region and ZNF384 plasmid into HEK293T cells upregulated NOL9, an effect diminished when binding site 2 was mutated (Fig. 4D). ChIP-qPCR further identified ZNF384 enrichment at the NOL9 promoter region (Fig. 4E and Supplementary Fig. 3B). In NOL9-overexpressing cells, ZNF384 knockdown reduced NOL9 expression (Fig. 4F and Supplementary Fig. 3C). Rescue experiments corroborated that ZNF384 knockdown could counteract NOL9’s gain-of-function, as cell proliferation was reduced (Supplementary Fig. 3D).

RiboSis gene expression is thought to be negatively correlated with patient drug response [6]. Our previous histochemical staining of clinical specimens found that NOL9 was upregulated in tumor tissues of HCC patients with sorafenib resistance. Therefore, we sought to verify the potential mechanism for NOL9 upregulation in sorafenib-resistant HCC. The sorafenib-resistant HepG2 cell line (Hep.G2-SR) was established through six months of sorafenib treatment. We found that NOL9 was also overexpressed in this cell line, and ZNF384 knockdown increased Hep.G2-SR’s response to sorafenib (Fig. 4G, H). These findings underscore ZNF384’s role in enhancing NOL9 expression and activating its promoter.

### Epigenetic regulation of NOL9 by DNA methylation

Transcription factors are pivotal in regulating the expression of DNA-methylated genes. Therefore, we further assessed methylation patterns across various NOL9 gene sites based on the TCGA-LIHC database. Using the UCSC Genome Bioinformatics Site, we identified a high enrichment of CpG islands within the NOL9 promoter region (Supplementary Fig. 3E), suggesting a potential relationship between NOL9 expression and DNA methylation. A correlation analysis revealed a significant inverse correlation between NOL9 transcriptional expression and cg09513309 methylation (Fig. 4I and Supplementary Fig. 3F). Previous KM survival analysis indicated that higher NOL9 expression correlated with poorer overall survival (OS) and progression-free interval (PFI) in three different HCC cohorts, whereas increased methylation at the NOL9 cg09513309 site was associated with improved prognosis (OS: P = 0.001; PFI: P = 0.018) (Supplementary Fig. 3G).

Our prior analysis suggested that NOL9 promoter methylation inversely correlated with its mRNA levels in HCC, indicating that methylation mediates the regulation of its expression [20]. We hypothesized that the methylation level of the NOL9 promoter decreases in HCC, particularly in sorafenib-resistant HCC. Using MSP, we observed reduced NOL9 methylation in both Hep.G2-SR and decitabine-treated cells, with decitabine having a more pronounced effect (Fig. 4J). To further explore the mechanism of methylation changes in the NOL9 promoter region, we transfected siRNAs targeting DNA methyltransferases (DNMTs) into Hep.G2-SR cells. Methylation PCR revealed that DNMT1 knockdown significantly decreased the ratio of methylated NOL9, and the proportion of unmethylated NOL9 increased with DNMT1 knockdown (Fig. 4K). Additionally, DNMT1 overexpression suppressed NOL9 expression in Hep.G2-SR, while DNMT1 knockdown elevated NOL9 expression in Hep.G2 (Fig. 4L). ChIP-qPCR assays demonstrated that ZNF384 enrichment at the NOL9 promoter region decreased after DNMT1 overexpression, while DNMT1 knockdown increased ZNF384 enrichment (Fig. 4M). These results suggest that DNMT1 plays a role in the epigenetic regulation of the NOL9 promoter, which is crucial for HCC cell resistance to sorafenib.

### NOL9-mediated cell proliferation is β-catenin dependent

**Fig. 5:**
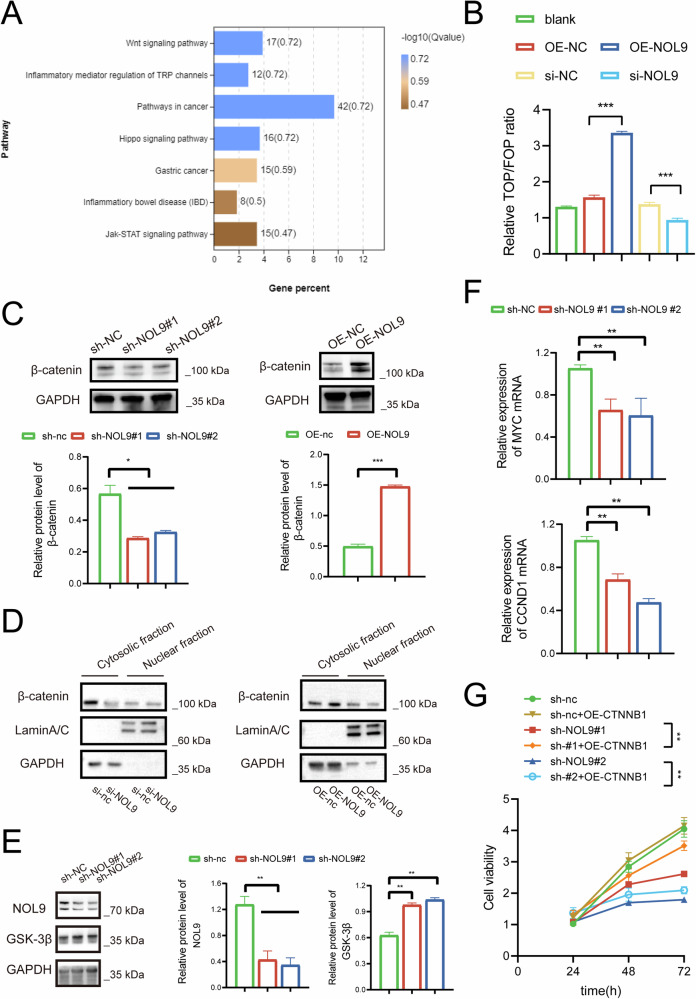
NOL9-mediated cell proliferation is β-catenin dependent. **A** KEGG enrichment analysis of DEGs obtained after NOL9 knockdown. **B** TOP/FOP-flash assays following NOL9 overexpression or knockdown. **C** Western blotting analysis of β-catenin expression following NOL9 overexpression or knockdown. **D** Nuclear-cytoplasmic fractionation analysis of β-catenin levels following NOL9 overexpression or knockdown. **E** Western blotting analysis of GSK-3β expression following NOL9 overexpression or knockdown. **F** RT-qPCR analysis of C-MYC and Cyclin D1 expression following NOL9 knockdown. **G** CCK8 analysis of cell proliferation following CTNNB1 overexpression in the Huh7 cell line stably silenced for NOL9.

To further investigate the molecular mechanism through which NOL9 regulates HCC cell proliferation, we performed RNA sequencing on NOL9-knockout HCC cells. KEGG enrichment analysis indicated that NOL9-mediated cell proliferation is dependent on the Wnt/β-catenin signaling pathway (Fig. 5A). This finding prompted us to assess the effect of NOL9 on the Wnt/β-catenin signaling pathway, and we evaluated the transcriptional activity of β-catenin/TCF after NOL9 overexpression or knockdown. Dual-luciferase assay results demonstrated that NOL9 increases the activity of the Wnt/β-catenin signaling pathway (Fig. 5B). We also analyzed the protein levels of key genes (β-catenin) in the Wnt/β-catenin signaling pathway. Western blotting revealed that NOL9 knockdown inhibited β-catenin protein expression, whereas overexpression of NOL9 had the opposite effect (Fig. 5C). Nuclear-cytoplasmic fractionation assays demonstrated that altering NOL9 expression does not significantly induce the nuclear translocation of β-catenin. However, it markedly affects β-catenin levels in the cytoplasm. NOL9 knockdown significantly reduced β-catenin levels in the cytoplasm, while overexpression of NOL9 notably increased them (Fig. 5D). Furthermore, western blotting revealed that glycogen synthase kinase 3β (GSK-3β), a key regulator of the Wnt/β-catenin signaling pathway, is modulated by NOL9. Specifically, NOL9 knockdown led to a significant increase in GSK-3β protein levels (Fig. 5E). C-MYC and Cyclin D1, critical downstream targets of the Wnt/β-catenin signaling pathway, play pivotal roles in cell cycle progression and proliferation. RT-qPCR analysis showed that NOL9 knockdown significantly reduced the mRNA levels of **MYC** and **CCND1** (Fig. 5F). These findings further underscore the role of NOL9 in regulating the Wnt/β-catenin signaling pathway and its downstream targets. Notably, overexpression of CTNNB1 in NOL9-downregulated stable strains promoted cell proliferation (Fig. 5G). Together, these results demonstrate that NOL9 activates the Wnt/β-catenin pathway in HCC cells, thereby promoting cell proliferation.

## Discussion

HCC represents a significant global health burden, with a complex interplay of genetic and environmental factors contributing to its pathogenesis. Despite advances in understanding its molecular underpinnings, effective therapeutic strategies remain elusive, necessitating a deeper exploration of the intricate regulatory networks driving HCC progression. Among the emerging candidates in HCC research is NOL9, a 5’-polynucleotide kinase primarily localized in the nucleus [19]. While its canonical role involves rRNA processing, recent studies have implicated NOL9 in various cellular processes, including tumorigenesis [21].

NOL9’s multifaceted involvement in cancer biology is underscored by its aberrant expression patterns in malignancies such as triple-negative breast cancer and acute myeloid leukemia [21, 22]. Previously, we found that elevated NOL9 expression is linked to tumor progression and poor prognosis in HCC, highlighting its potential as a prognostic marker and therapeutic target [20]. However, the underlying mechanisms driving NOL9 dysregulation and its functional significance in HCC pathogenesis remain incompletely understood. Building upon this foundation, this study explores the functional significance of NOL9 in HCC progression and its interaction with epigenetic regulators and signaling pathways.

Our study begins by utilizing the TCGA-LIHC dataset, our institution’s HCC database, and biochemical microarray database, which includes a cohort of 517 patients. We demonstrate that NOL9 overexpression correlates with poor prognosis in HCC patients, highlighting its potential as a prognostic biomarker. Through comprehensive clinical analyses and the construction of a nomogram, we establish NOL9 as a key determinant of HCC patient survival, providing valuable insights for risk stratification and treatment decisions. Mounting evidence highlights the dysregulation of key components in ribosome biosynthesis, including ribosomal proteins [23], rRNA processing enzymes [24], and assembly factors [25], as precursors to heightened malignant potential. Indeed, dysregulated ribosome biogenesis has emerged as a hallmark of therapeutic resistance in various cancer models [26]. Our study reveals NOL9’s pivotal role in driving proliferation, suppressing apoptosis, and fostering resistance to sorafenib both in vivo and in vitro.

Ribosome biosynthesis, a complex and energy-intensive process, is closely intertwined with the cell cycle, ensuring coordinated cellular growth and proliferation [27]. Through KEGG and GO analysis, we found that NOL9 is correlated with cell cycle regulation, potentially influencing processes such as chromosome segregation, nuclear division, DNA replication, and cell cycle checkpoints. Flow cytometry further verifies that NOL9 facilitates the G1/S cell cycle transition. During the G1 phase, key regulators of ribosome biogenesis, including transcription factors like c-Myc and RNA polymerases, are activated to promote the transcription of rRNA and ribosomal protein genes [28, 29]. As cells progress into the S phase, rRNA genes are transcribed by RNA polymerase I, producing precursor rRNA transcripts that undergo processing and modification to generate mature 18S, 5.8S, and 28S rRNAs, which then assemble with ribosomal proteins to form the ribosome’s small and large subunits [9]. NOL9, as a key participant in pre-rRNA cleavage and 28S rRNA maturation, is essential for the cell cycle, and its dysregulation may lead to genomic instability, impaired proliferation, and ultimately, disease states such as cancer.

Epigenetic dysregulation, particularly DNA methylation, has emerged as a crucial mechanism in cancer development, influencing gene expression patterns and cellular phenotypes [30]. The dynamic interplay between DNA methylation and transcription factor binding modulates gene expression, shaping the molecular landscape of tumors [31]. In HCC, aberrant DNA methylation patterns contribute to tumor heterogeneity and therapeutic resistance, highlighting their clinical significance [32]. Our findings further elucidate the complex regulatory network governing NOL9 expression in HCC. We identify DNA methylation, primarily mediated by DNMT1, as a critical epigenetic regulator of NOL9 expression. Notably, the interaction between NOL9 and ZNF384 highlights a multifaceted regulatory axis, which may provide new insights into NOL9’s oncogenic role in HCC.

Additionally, the Wnt/β-catenin signaling pathway has garnered considerable attention in HCC due to its pivotal role in cell proliferation, differentiation, and survival [33]. Dysregulated Wnt/β-catenin signaling is a hallmark feature of HCC, driving tumor progression and contributing to therapeutic resistance [34, 35]. β-catenin activity is tightly regulated by multiple mechanisms, including protein stability via the ubiquitin-proteasome and autophagy pathways, subcellular localization mediated by nuclear pore complexes and chaperones, and transcriptional activity orchestrated by transcriptional complexes and regulatory cofactors [36, 37]. GSK-3β, a key kinase within the β-catenin degradation complex, phosphorylates β-catenin, targeting it for ubiquitination and proteasomal degradation, thereby maintaining β-catenin protein homeostasis [38, 39]. Our findings reveal a novel role for NOL9 in modulating the Wnt/β-catenin signaling pathway by influencing β-catenin stability and cytoplasmic accumulation through GSK-3β regulation. Specifically, NOL9 appears to enhance β-catenin stability by downregulating GSK-3β levels, which may reduce β-catenin degradation. This positions NOL9 as a potential oncogenic driver in HCC by contributing to the aberrant activation of the Wnt/β-catenin axis. By elucidating the link between NOL9 and the Wnt/β-catenin signaling pathway, our study provides valuable insights into the molecular mechanisms driving HCC progression. These findings underscore the therapeutic potential of targeting the NOL9-Wnt/β-catenin axis, particularly in HCC cases characterized by dysregulated β-catenin signaling. Future research aimed at further dissecting this axis could inform the development of novel targeted therapies to combat HCC.

However, while our study provides valuable insights into the role of NOL9 in HCC, several questions remain unanswered. Future investigations should explore the broader regulatory networks and downstream effectors of NOL9 to fully understand its functions in HCC pathogenesis. Additionally, the therapeutic implications of targeting NOL9 and its associated pathways warrant further exploration, with potential applications in overcoming therapeutic resistance and improving patient outcomes in HCC.

In summary, our findings not only support previous observations of NOL9’s oncogenic role but also reveal novel insights into its regulation and downstream effects in HCC. Furthermore, our exploration of ribosome biogenesis as a therapeutic target in cancer emphasizes the broader impact of these findings, offering new avenues for improving treatment strategies against HCC and other cancers.

## Conclusion

In conclusion, our study demonstrates that NOL9, which is upregulated in HCC tissues, drives tumor progression and influences resistance to sorafenib. Mechanistically, NOL9 expression is regulated by DNA methylation and ZNF384, and it promotes HCC cell proliferation through a β-catenin-dependent pathway. These findings highlight NOL9’s potential as a diagnostic biomarker and therapeutic target for improving HCC management.

## Supplementary information

Supplementary Materials

Western Blotting

## Data Availability

All data supporting the findings of this study are available from the corresponding author upon reasonable request. Correspondence and requests for materials should be addressed to Chan Xie.
