# Supplementary material for: Nucleolar NOL9 regulated by DNA methylation promotes hepatocellular carcinoma growth through activation of Wnt/β-catenin signaling pathway
Source: Cell Death Dis. 2025 Feb 15;16(1):100. doi: 10.1038/s41419-025-07393-7 (PMC11830072; doi:10.1038/s41419-025-07393-7)
Supplement: Supplementary file 1 — Supplementary Materials [file 41419_2025_7393_MOESM1_ESM.pdf]

Nucleolar NOL9 regulated by DNA methylation promotes hepatocellular carcinoma growth by activating wnt/β-catenin pathway

1. Materials and Methods

1.1 Data Download: The TCGA-LIHC dataset, comprising 524 samples (374 cancer and 50 normal samples), was downloaded from the UCSC Xena database (https://xenabrowser.net/datapages/). The dataset includes gene expression data (log2(FPKM+1)), clinical information, and survival data.

1.2 ROC Curve: The R package “PROR” was used to plot the ROC curve for the NOL9 gene.

1.3 Univariate and Multivariate Cox Regression: Clinical variables, including age, metastasis (M), node status (N), tumor size (T), gender, and stage, were used to group the samples. Wilcoxon rank-sum tests were conducted to assess the significance of these differences between groups. Univariate and multivariate Cox regression analyses were performed using the TCGA database to determine whether NOL9 is an independent prognostic factor. A nomogram was constructed based on gene expression levels and clinical pathological parameters from the TCGA database to estimate the potential 1-year, 3-year, and 5-year survival rates of liver hepatocellular carcinoma (LIHC) patients. Calibration curves for the nomogram were also created to assess the deviation between predicted and actual survival rates. Utilizing the multivariate analysis results, a nomogram incorporating NOL9 gene expression, T, and M data was developed to estimate 1-year, 3-year, and 5-year survival probabilities for hepatocellular carcinoma (HCC) patients, and calibration curves were generated to assess agreement between predicted and observed survival.

2. Supplementary Figure Legends

Supplementary Fig. 1:

A. Correlation of clinical characteristics and NOL9 expression in the TCGA-LIHC cohort.

B. Diagnostic values of NOL9 in HCC.

C. Univariate and multivariate Cox analysis.

D-E. A nomogram and calibration curve of the nomogram.

F. Western blotting and RT-qPCR analysis of NOL9 expression in seven paired HCC and tumor-adjacent normal tissue samples, with GAPDH used as a loading control.

Supplementary Fig. 2:

A. Western blotting and RT-qPCR analysis of NOL9 expression in the indicated cells.

B-C. Establishment of the Huh7 cell line with stable NOL9 knockdown, and the establishment of the HepG2 cell line with high NOL9 expression. Western blotting and RT-qPCR analysis of NOL9 expression.

D. Quantification of pre-rRNA and 28S rRNA levels following NOL9 overexpression or knockdown.

Supplementary Fig. 3:

A. RT-qPCR analysis of NOL9 expression, with GAPDH used as a loading control.

B. ChIP assay showing the enrichment of ZNF384 at the NOL9 promoter region.

C. RT-qPCR analysis of NOL9 expression, with GAPDH used as a loading control.

D. CCK8 assay results.

E. Methylation loci in the NOL9 gene.

F. Methylation levels at different loci in the NOL9 gene.

G. Overall survival is higher in HCC patients with high cg09513309 methylation; progression-free interval is also higher in patients with high cg09513309 methylation.

3. Supplementary Table

Supplementary Table 1: The Reagents used in this project.

REAGENT	SOURCE	Catalog
Sorafenib	MedChemExpress	HY-10201
5-Aza-2'-deoxycytidine	MedChemExpress	HY-A0004
Cell Counting Kit-8 assays	DOJINDO	CK04
Thymidine Analog 5-ethynyl-2'-deoxyuridine Assays	KeyGEN	KGA337
Crystal violet	Beyotime	C0121
NOL9 antibody for IH	absin	abs139241
NOL9 antibody for WB	abcam	ab140597
GAPDH antibody for WB	abcam	ab8245
β-Tubulin antibody for WB	ABclonal	A12289-50
Rb antibody for WB	CST	9309S
P-Rb antibody for WB	CST	8516T
CDK6 antibody for WB	ThermoFisher	PA5-27978
Cyclin D1 antibody for WB	CST	2978T
β-catenin antibody for WB	CST	9562S
Goat anti-rabbit IgG-HRP	saiguotech	E030120-01
Goat anti-mouse IgG-HRP	saiguotech	E030110-01
ZNF384 antibody for ChIP	abcam	ab176689
RIPA lysis buffer	EpiZyme	PC101
Polyvinylidene fluoride membranes	Millipore	IPVH00010
Excellent Chemiluminescent Substrate detection kit	EpiZyme	SQ201
RNA Quick Purification kit	ES Science	RN001

Fast Reverse Transcription kit (5 × Mix with gDNA Remover)	ES Science	RT001
TB Green Premix Ex Taq II	Takara	RR820A
Lipofectamine 3,000	Invitrogen	SQ201
Puromycin	InvivoGen	Ant-pr-5b
EpiTect Bisulfite Kits	Qiagen	59104
Annexin V-APC/PI Apoptosis Detection Kit	KeyGEN	KGA1030-100
Cell Cycle Detection Kit	KeyGEN	KGA512
Dual-Luciferase Reporter Assay System	Promega	E1910
Nuclear and Cytoplasmic Extraction Reagents Kit	ThermoFisher	78833

Supplementary Table 2: Primer sequences for quantitative real-time PCR.

Gene name	Primer
	RT-qPCR
NOL9-F	5'- ATCTGTCGTGTGACTTGCCTC -3'
NOL9-R	5'- AAAAATTCTGCATCGACCAACG -3'
GAPDH-F	5'- ACAACTTTGGTATCGTGGAAGG -3'
GAPDH-R	5'- GCCATCACGCCACAGTTTC -3'
MYC-F	5'- GGCTCCTGGCAAAAGGTCA -3'
MYC-R	5'- CTGCGTAGTTGTGCTGATGT -3'
CCND1-F	5'- GCTGCGAAGTGGAAACCATC -3'
CCND1-R	5'- CCTCCTTCTGCACACATTTGAA -3'
Pre-rRNA-F	5'- GAAACCTTCCGACCCCTCTC -3'
Pre-rRNA-R	5'- AGTGCGTTCGAAGTGTCGAT -3'
28S rRNA-F	5'- GCAACAAGGAAAAGAACAACGACC -3'
28S rRNA-R	5'- GGTGACGTTTGTATGGCACTAAA -3'
	ChIP
ChIP-NOL9-F	5'- CATGAGAATCACTTGAGCCCAG -3'
ChIP-NOL9-R	5'- GTTCAAAGTTCCCTAGAGTCCCAG -3'
	MSP
MSP-NOL9-methylated-F	5'- GTTTTATTTAGGAATTGGGTAGGAC -3'
MSP-NOL9-methylated-R	5'- CAAATAAAAAATTCTAAAACCTCGC -3'
MSP-NOL9-unmethylated-F	5'- TTTATTTAGGAATTGGGTAGGATGA -3'
MSP-NOL9-unmethylated-R	5'- AAATAAAAAATTCTAAAACCTCACA -3'

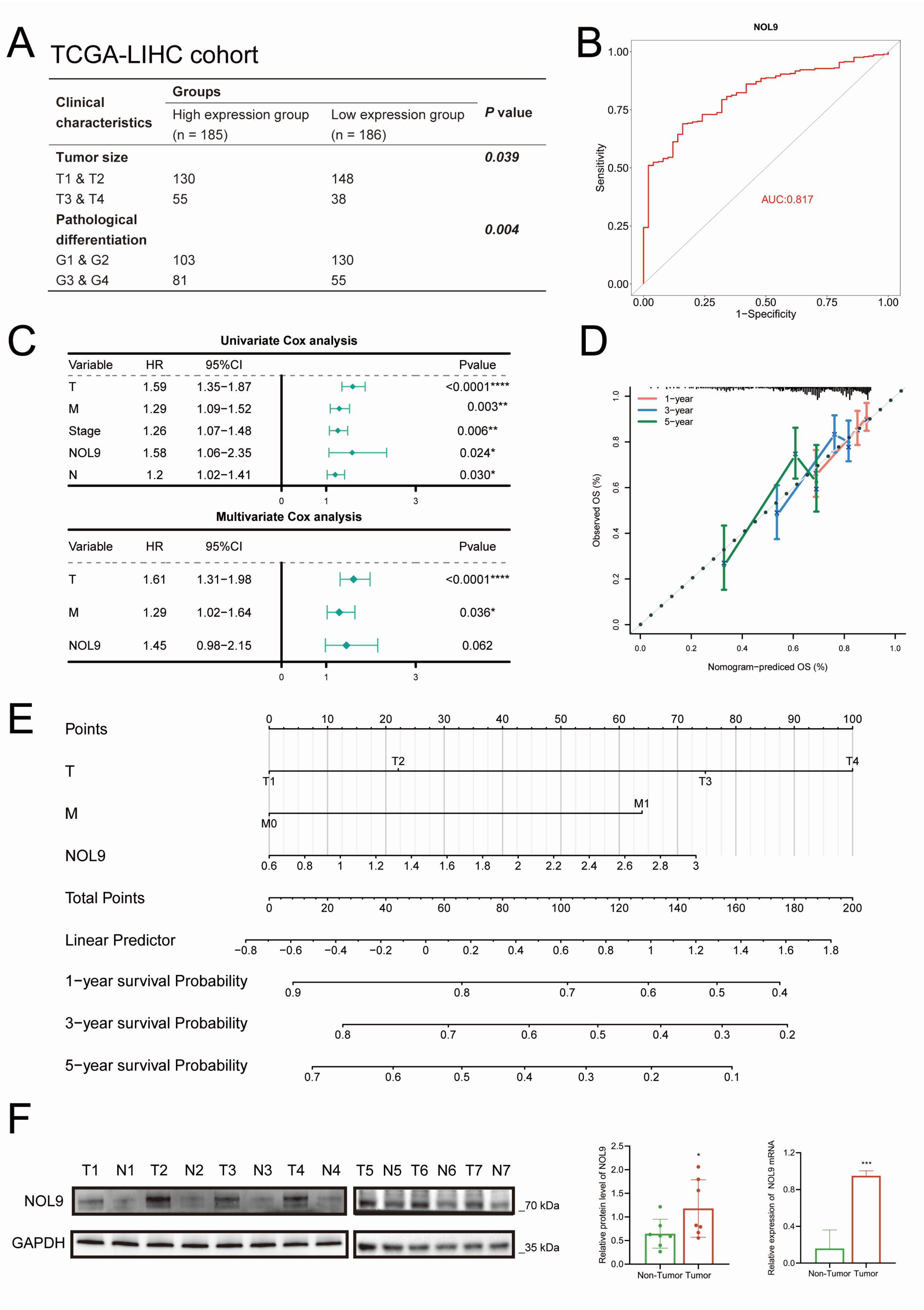

A TCGA-LIHC cohort

Clinical characteristics	Groups: High expression group (n = 185)	Groups: Low expression group (n = 186)	*P* value
**Tumor size**			***0.039***
T1 & T2	130	148	
T3 & T4	55	38	
**Pathological differentiation**			***0.004***
G1 & G2	103	130	
G3 & G4	81	55	

C Univariate Cox analysis

Variable	HR	95%CI	Pvalue
T	1.59	1.35−1.87	<0.0001****
M	1.29	1.09−1.52	0.003**
Stage	1.26	1.07−1.48	0.006**
NOL9	1.58	1.06−2.35	0.024*
N	1.2	1.02−1.41	0.030*

Multivariate Cox analysis

Variable	HR	95%CI	Pvalue
T	1.61	1.31−1.98	<0.0001****
M	1.29	1.02−1.64	0.036*
NOL9	1.45	0.98−2.15	0.062

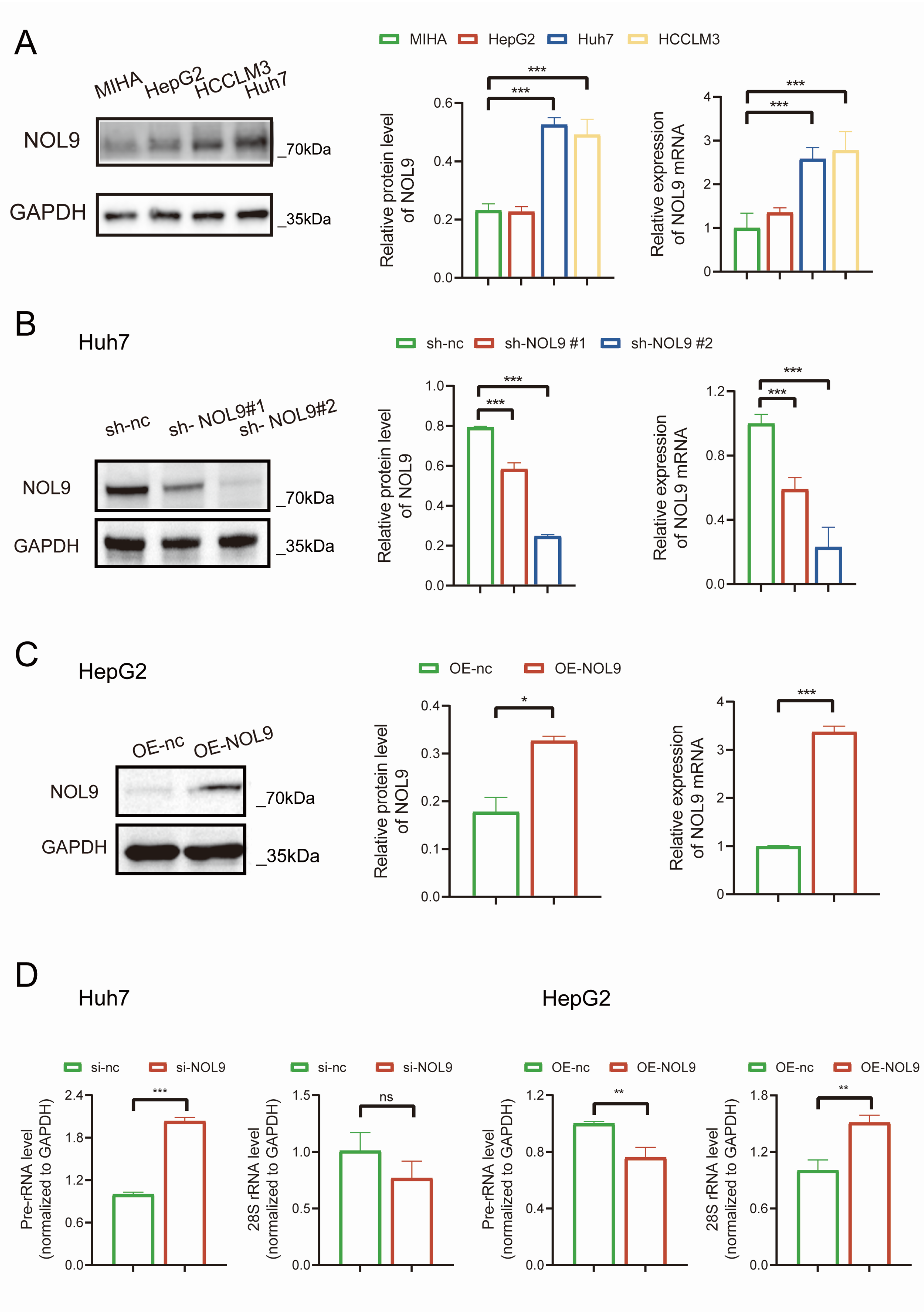

A
MIHA
HepG2
HCCLM3
Huh7
NOL9
_70kDa
GAPDH
_35kDa
MIHA HepG2 Huh7 HCCLM3
Relative protein level of NOL9
Relative expression of NOL9 mRNA
***
B
Huh7
sh-nc
sh- NOL9#1
sh- NOL9#2
NOL9
_70kDa
GAPDH
_35kDa
sh-nc sh-NOL9 #1 sh-NOL9 #2
Relative protein level of NOL9
Relative expression of NOL9 mRNA
***
C
HepG2
OE-nc
OE-NOL9
NOL9
_70kDa
GAPDH
_35kDa
OE-nc OE-NOL9
Relative protein level of NOL9
Relative expression of NOL9 mRNA
*
***
D
Huh7
HepG2
si-nc si-NOL9
Pre-rRNA level (normalized to GAPDH)
28S rRNA level (normalized to GAPDH)
OE-nc OE-NOL9
***
ns
**

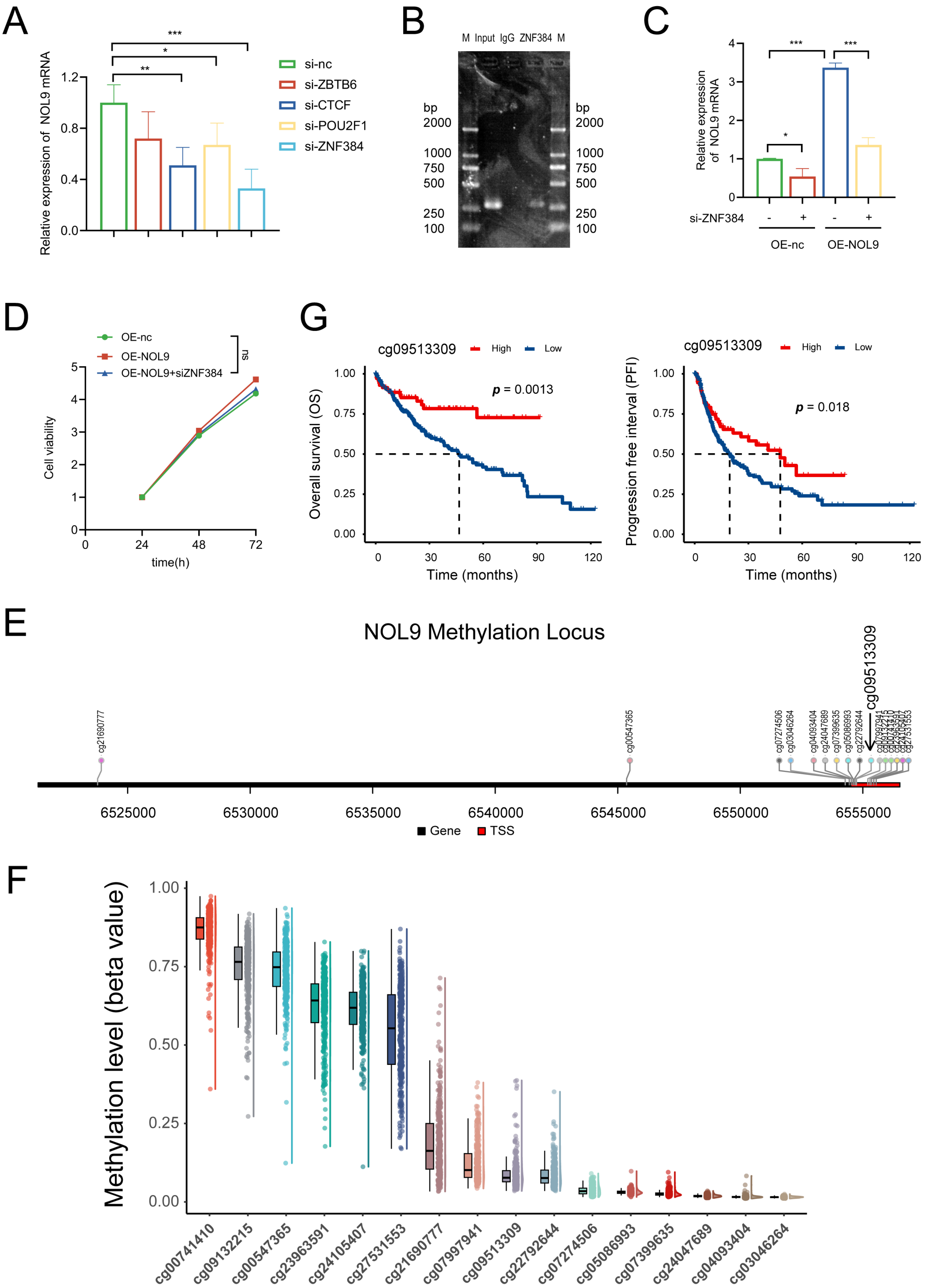
A
Relative expression of NOL9 mRNA
si-nc
si-ZBTB6
si-CTCF
si-POU2F1
si-ZNF384
B
M Input IgG ZNF384 M
bp
2000
1000
750
500
250
100
C
Relative expression of NOL9 mRNA
si-ZNF384
OE-nc
OE-NOL9
D
OE-nc
OE-NOL9
OE-NOL9+siZNF384
ns
Cell viability
time(h)
G
cg09513309 High Low
Overall survival (OS)
p = 0.0013
Time (months)
Progression free interval (PFI)
p = 0.018
E
NOL9 Methylation Locus
cg09513309
Gene
TSS
F
Methylation level (beta value)
cg00741410
cg09132215
cg00547365
cg23963591
cg24105407
cg27531553
cg21690777
cg07997941
cg09513309
cg22792644
cg07274506
cg05086993
cg07399635
cg24047689
cg04093404
cg03046264
