# Supplementary material for: Nucleolar NOL9 regulated by DNA methylation promotes hepatocellular carcinoma growth through activation of Wnt/β-catenin signaling pathway
Source: Cell Death Dis. 2025 Feb 15;16(1):100. doi: 10.1038/s41419-025-07393-7 (PMC11830072; doi:10.1038/s41419-025-07393-7)

Original western blots - Figure I

Figure 2F

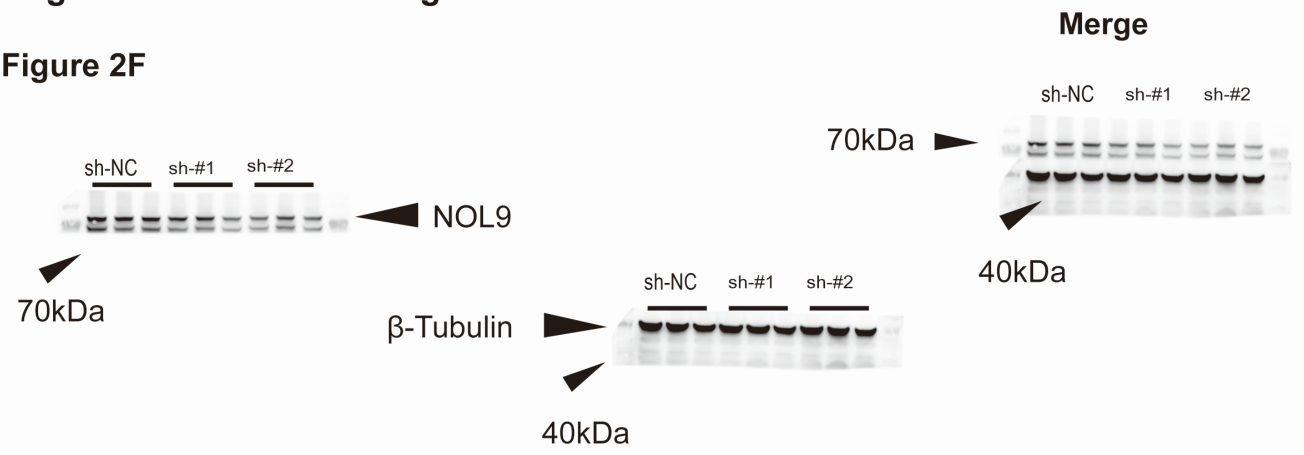

Figure 3E

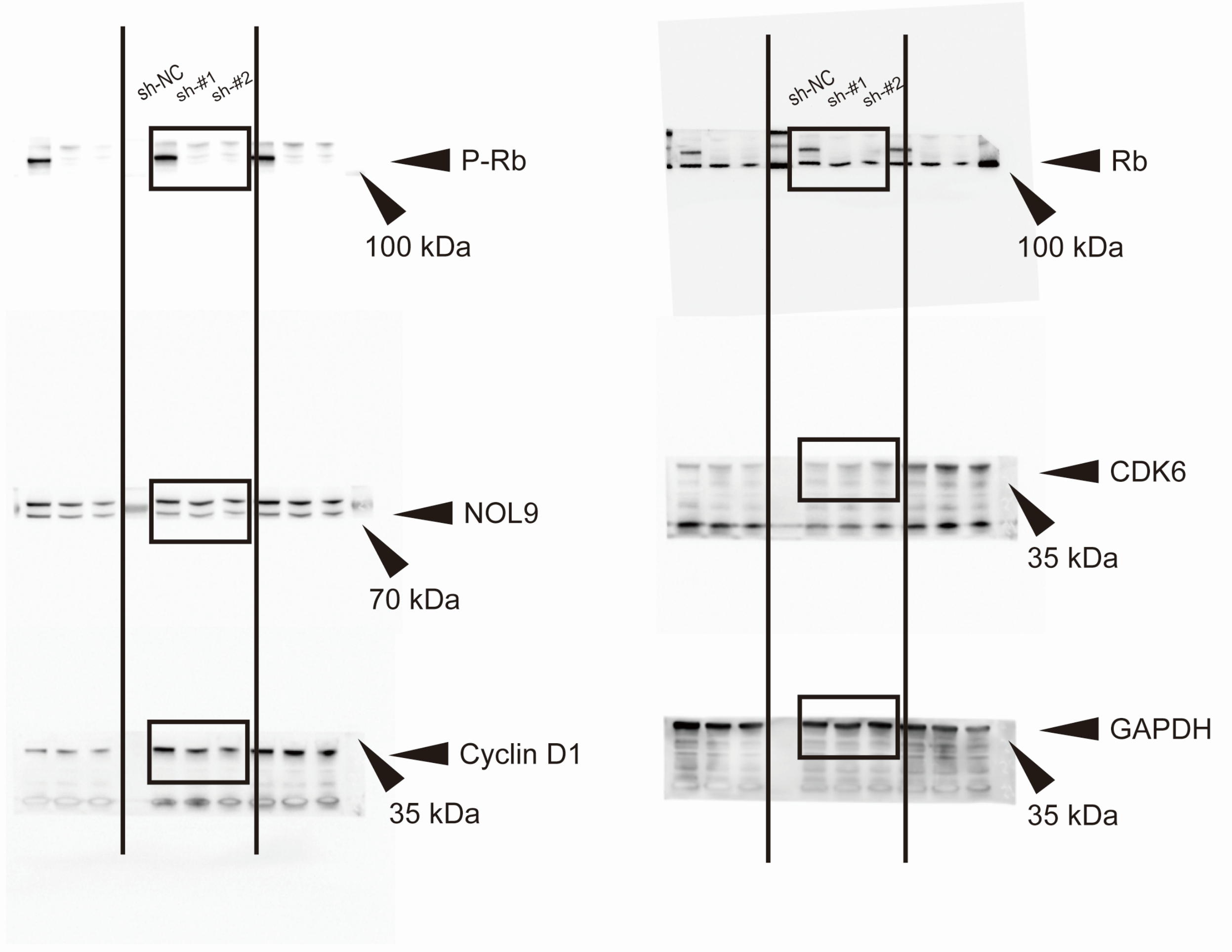

Figure 4A

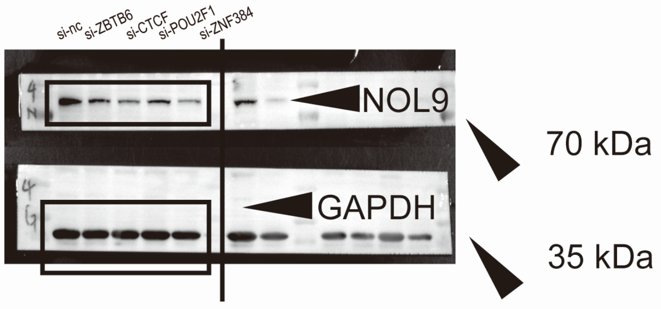

Original western blots - Figure II

Figure 4F

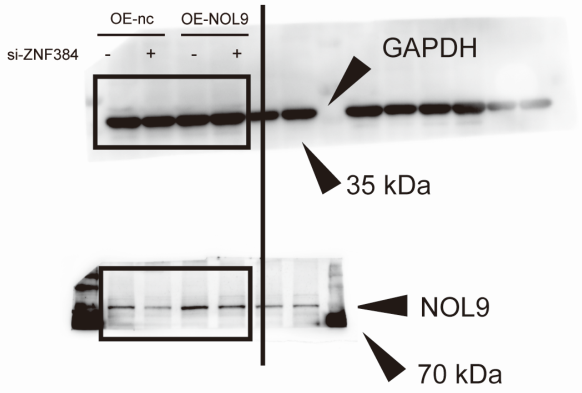

Figure 4G

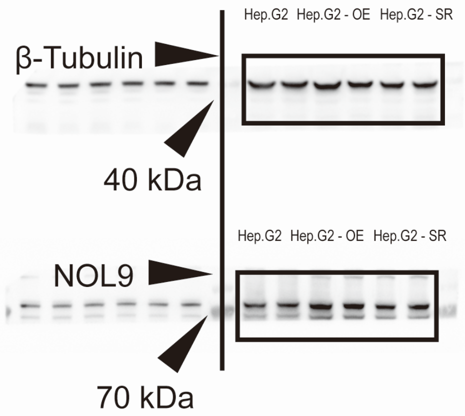

Figure 5C

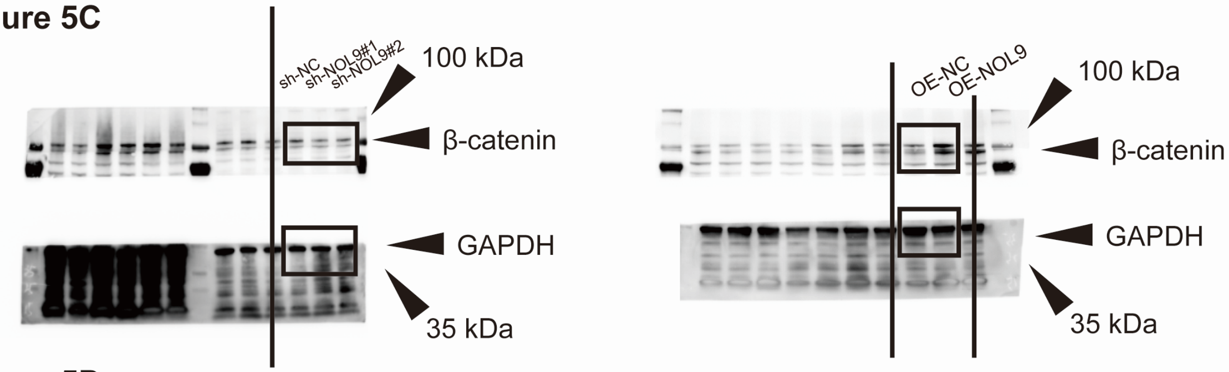

Figure 5D

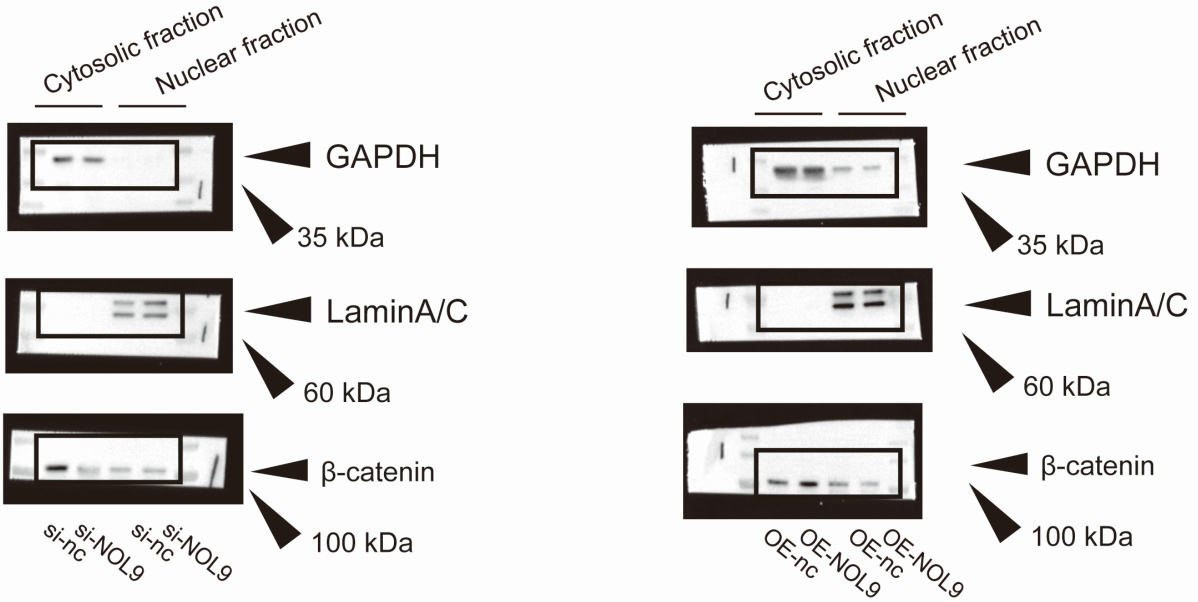

Figure 5E

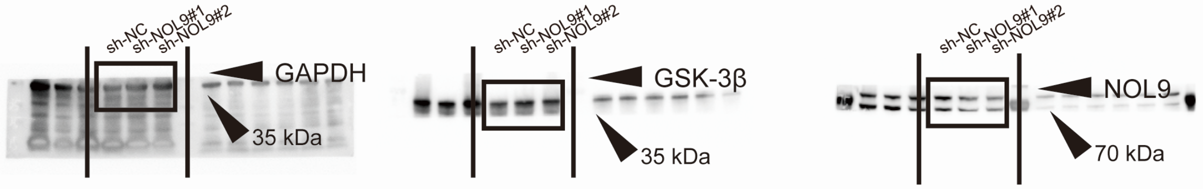

Original western blots - Supplementary Figure

Supplementary Figure 1F

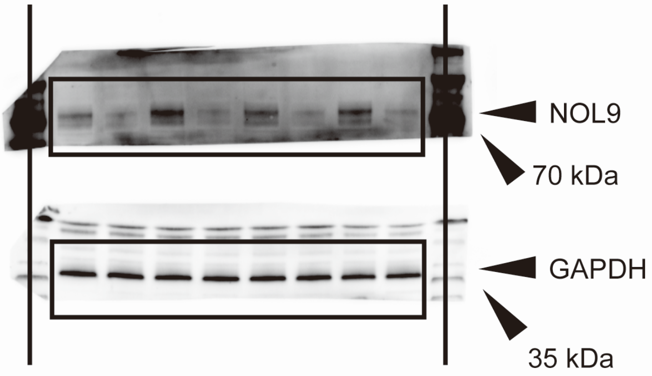

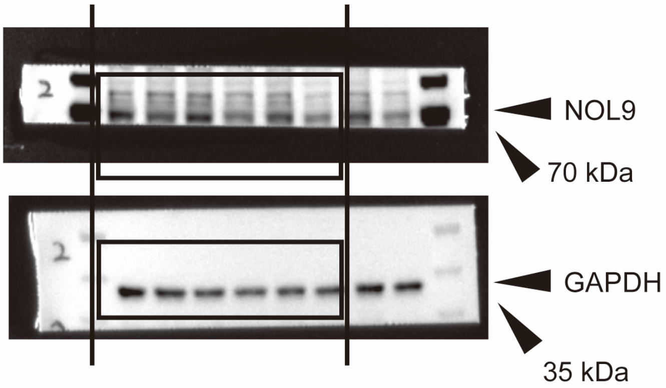

Supplementary Figure 2A

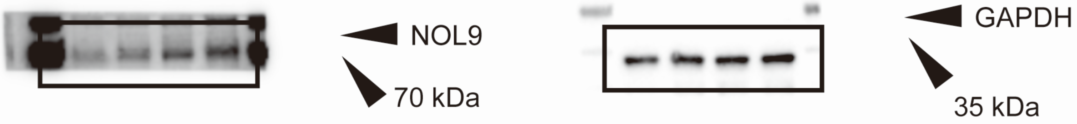

Supplementary Figure 2B

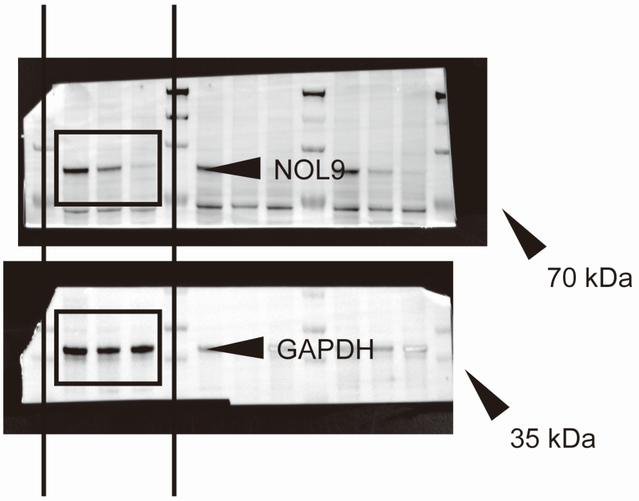

Supplementary Figure 2C

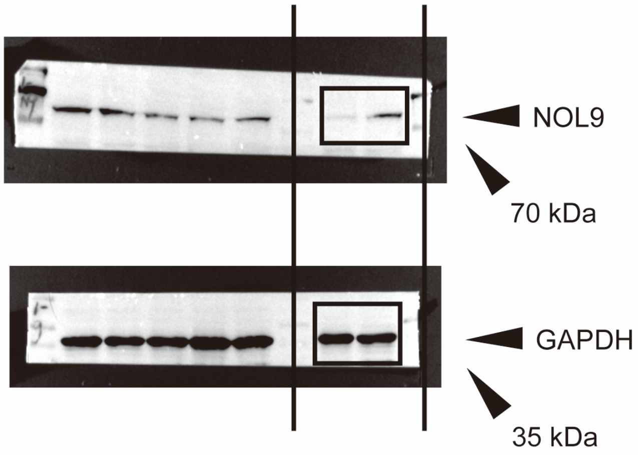

Supplement: Supplementary file 2 — Western Blotting [file 41419_2025_7393_MOESM2_ESM.pdf]
